# Mice Lacking Pten in Osteoblasts Have Improved Intramembranous and Late Endochondral Fracture Healing

**DOI:** 10.1371/journal.pone.0063857

**Published:** 2013-05-13

**Authors:** Travis A. Burgers, Martin F. Hoffmann, Caitlyn J. Collins, Juraj Zahatnansky, Martin A. Alvarado, Michael R. Morris, Debra L. Sietsema, James J. Mason, Clifford B. Jones, Heidi L. Ploeg, Bart O. Williams

**Affiliations:** 1 Center for Skeletal Disease Research, Van Andel Research Institute, Grand Rapids, Michigan, United States of America; 2 Grand Rapids Medical Education Partners, Grand Rapids, Michigan, United States of America; 3 Trauma Center, Orthopaedic Associates of Michigan, Grand Rapids, Michigan, United States of America; 4 Department of Mechanical Engineering, University of Wisconsin-Madison, Madison, Wisconsin, United States of America; 5 Creston High School, Grand Rapids, Michigan, United States of America; 6 Grand Rapids Area Pre-College Engineering Program, Grand Rapids, Michigan, United States of America; 7 College of Human Medicine, Michigan State University, Grand Rapids, Michigan, United States of America; INSERM U1059/LBTO, Université Jean Monnet, France

## Abstract

The failure of an osseous fracture to heal (development of a non-union) is a common and debilitating clinical problem. Mice lacking the tumor suppressor Pten in osteoblasts have dramatic and progressive increases in bone volume and density throughout life. Since fracture healing is a recapitulation of bone development, we investigated the process of fracture healing in mice lacking Pten in osteoblasts (*Ocn-cre^tg/+;^Pten^flox/flox^*). Mid-diaphyseal femoral fractures induced in wild-type and *Ocn-cre^tg/+;^Pten^flox/flox^* mice were studied via micro-computed tomography (µCT) scans, biomechanical testing, histological and histomorphometric analysis, and protein expression analysis. *Ocn-cre^tg/+;^Pten^flox/flox^* mice had significantly stiffer and stronger intact bones relative to controls in all cohorts. They also had significantly stiffer healing bones at day 28 post-fracture (PF) and significantly stronger healing bones at days 14, 21, and 28 PF. At day 7 PF, the proximal and distal ends of the Pten mutant calluses were more ossified. By day 28 PF, Pten mutants had larger and more mineralized calluses. Pten mutants had improved intramembranous bone formation during healing originating from the periosteum. They also had improved endochondral bone formation later in the healing process, after mature osteoblasts are present in the callus. Our results indicate that the inhibition of Pten can improve fracture healing and that the local or short-term use of commercially available Pten-inhibiting agents may have clinical application for enhancing fracture healing.

## Introduction

Fracture healing is a complex process involving several overlapping stages: inflammation, formation of soft and hard callus, and bone remodeling [1]. As a recapitulation of embryonic skeletal development [2], fracture repair follows a one-way path of similarly regulated chondrogenic and osteoblastic phases of bone formation leading toward bone healing and remodeling. Most fractures heal by a combination of endochondral and intramembranous ossification [3]. In endochondral ossification, bone formation occurs from a cartilaginous template [4]. In intramembranous ossification, bone forms directly from the cortical bone and periosteum to bridge the fracture gap [4], but it is rare for a fracture to heal exclusively by this mechanism [3]. It is estimated that 10–20% of fractures do not heal in a timely manner [5], [6]. The increased costs for treatment of impaired healing (non-union) and the increased morbidity for the individual patients [7] makes enhancing the process of fracture healing an important priority.

Skeletal development is regulated by morphogens (e.g., bone morphogenic proteins) [8] and growth factors (e.g., insulin-like growth factor 1) [9]. These growth factors regulate osteoblast differentiation, maturation, and survival through the anti-apoptotic pathways [10]. The lipid kinase phosphatidylinositol 3-kinase (PI3K) is involved in these pathways [11]. PI3K phosphorylates PI(4)P or PI(4,5)P_2_ to produce the second messengers PI(3,4)P_2_ and PI(3,4,5)P_3_ (known as PIP_3_) [11]. A major downstream target of PIP_3_ is Akt. PIP_3_ recruits Akt and PI-dependent kinase 1 (PDK1), and then PDK1 phosphorylates Akt, which causes it to be activated [11]. Activated Akt promotes cell growth, proliferation, survival, and metabolism [12], and regulates pathways related to GSK3 [11], BAD/Bcl-XL [11], p53 [13], and mTOR [14].

The *PTEN* gene (phosphatase and tensin homologue deleted on human chromosome 10) encodes a PIP phosphatase that dephosphorylates PIP_3_ and negatively regulates activation induced by PI3K. PTEN is deleted or inactivated in various types of tumors [15]. PTEN blocks the activation of Akt, affecting cell cycling, translation, and apoptosis [15]. When PTEN function is blocked, PIP_3_ accumulates and Akt is continually activated and results in increased in cell proliferation, survival, and migration [16]. Mice carrying a Cre-mediated osteoblast-specific deletion of the *Pten* gene (*Ocn-cre^tg/+;^Pten^flox/flox^*) had normal body size but showed dramatic and progressive increases in bone volume and density throughout life [17]. Osteoblasts from such mutant mice were less susceptible to apoptosis and had accelerated differentiation capacity [17]. These findings provide in vivo evidence that signaling via the PI3K/Pten pathway promotes osteoblast survival and function. Based on these findings, we hypothesize that inhibition of Pten in osteoblasts will lead to improved and more rapid fracture healing.

## Methods

This study was approved by the Institutional Animal Care and Use Committee at the Van Andel Research Institute. Mice lacking Pten in osteoblasts (*Ocn-cre^tg/+;^Pten^flox/flox^*) and wild-type controls (*Ocn-cre^+/+;^Pten^flox/flox^*) mice were generated [17]. To examine fracture healing and biomechanical characteristics, surgery was performed on 126 mice that were 11–12 weeks old following an established procedure [18]. Each animal was anesthetized using a subcutaneous weight-matched dose of tribromoethanol (average 350 µL dose of 0.079 mg/µL solution; Avertin; Winthrop Laboratories, New York). The fur on the right leg was removed and the patella was moved laterally to expose the distal femur. A 25-gauge needle was inserted retrograde in the femoral canal at the intercondylar notch and was advanced until it reached the cortex of the proximal femur. The needle was cut at the distal end with minimal distal intraarticular protrusion and was positioned like an intramedullary nail is in human long bone fractures [19]. This needle will not deter osteoblasts from forming bone in the callus because osteoblast progenitors enter the callus with invading blood vessels [20]. The patella was relocated and the knee arthrotomy was closed with a 6.0 silk suture to maintain patella positioning. The skin was closed with a surgical staple. Standardized radiographic imaging (PixArray 100, Bioptix Inc., Tucson, Arizona) was performed using standardized settings (approximately 31 kV for 9 s) to confirm needle positioning. A right mid-diaphyseal femoral fracture was created using a blunt impact force in a validated three-point bending technique [18]. Standardized radiographic imaging confirmed fracture location and type. Postoperative pain medication was managed with subcutaneous doses of tramadol [21] (20 mg/kg; Sigma-Aldrich, St. Louis, MO) administered at the time of surgery and at 12, 24, and 36 h after fracture.

Mice were divided into three groups: biomechanical and µCT evaluation (*n* = 66), histological evaluation (*n* = 30), and protein expression (*n* = 30). For the biomechanical and µCT evaluation, 45 fractured femurs and 66 intact, contralateral femurs and were used; 21 fractured femurs (10 wild-type and 11 Pten mutant animals) were excluded because the fracture was oblique, comminuted or incomplete, as determined by follow-up radiographs. Thirty mice were used for histological evaluation, and representative samples were chosen. Mice were sacrificed at 7, 14, 21, and 28 d PF.

### Biomechanical and µCT Evaluation

Both the fractured and intact femurs were excised and cleaned of the surrounding soft tissue. The intramedullary needles in the fractured femurs were removed and samples were stored at –20°C in saline-saturated gauze. The fractured and contralateral control femurs were scanned in saline by µ-computed tomography (µCT) using a Skyscan 1172 high-resolution micro-CT (Skyscan, Kontich, Belgium) with a voxel size of 13.3 µm. The two Skyscan calibration phantoms were included in each scan. Images were reconstructed using the Skyscan software.

In a repeated series of steps similar to methods in previous studies [22], [23], each phantom and femur was segmented into separate masks and made into three-dimensional volumes (Mimics x64 14.11, Materialise, Ann Arbor, MI; Figure S1a). Global thresholding based on Hounsfield unit (HU) was used to differentiate the cortical bone from the surrounding saline. The “3D LiveWire” tool was used to supplement the thresholding to differentiate the boundary of the callus from the saline. The “Region Growing” tool was used to separate the fractured and intact limbs from the thresholded mask, and a Boolean addition was made of the thresholded and 3D LiveWire regions for the fractured limb. The “Calculate Polylines” tool was used to draw polylines around the Boolean addition mask for the fractured limb and the region growing mask for the intact limb, and the “Cavity Fill From Polylines” tool was used so that no voxels were excluded from the volume of each segmented bone. The masks were cropped to include a uniform length of the bone from the intersection of the femoral neck and the greater trochanter to the intersection of the patellar groove and the diaphysis.

The volume and average HU of the masks was measured using Mimics. The linear relationship between the bone mineral content and HU [24], [25] of each scan was calculated from the known density of the two calibration phantoms (0.25 g/cm^3^ and 0.75 g/cm^3^) and their segmented HU values. This relationship was used to calculate the mineral density of the segmented fractured femur mask and intact femur. The mineral content of the fractured femur and the intact femur was found by multiplying each mask density by mask volume. The callus volume was calculated as the volume of the fractured femur (bone and callus) minus the volume of the intact femur (only bone), as shown in Figure S1b. The callus mineral content was calculated using the same method. The callus density was calculated as the callus mineral content divided by the callus volume.

For BV/TV analysis, five transverse slices were acquired: three in the central callus and two at the ends. One slice was taken at the center of the largest portion of callus and two more 0.5 mm proximal and distal to the determined center. The other two slices were taken at the proximal and distal ends of the callus. Using both sagittal and coronal cross-sections, the positions of the proximal and distal ends of the callus were determined by locating the point at which the edge of the callus intersected the intact portion of the fractured limb. A slice 1.0 mm distal to the proximal end of the callus and a slice 1.0 mm proximal to the distal end of the callus were used to characterize the amount of bone tissue at the proximal and distal ends of the callus, respectively.

The Digital Imaging and Communications in Medicine (DICOM) standard data files of the transverse slices were analyzed for mature callus area at a threshold of 600 mg/cm^3^ [26] using the “slice geometry” macro of BoneJ [27], a bone image analysis plugin for ImageJ (ImageJ 1.46, National Institutes of Health, Bethesda, MD). The mature (bone volume; BV) and total callus volume (TV) of each slice was measured and averaged for each region; BV/TV was calculated for each region. The central slices for day 7 were excluded because there was little to no endochondral ossification in groups at that time point.

For biomechanical assessment of the bones, all femurs were removed from the freezer, rehydrated in saline, and allowed to equilibrate to room temperature for at least 30 minutes. Four-point bending mechanical testing was performed on the right femur at a rate of 0.005 mm/s with a TestResources 570 L axial-torsional testing system (TestResources, Shakopee, MN). The distances between the lower and upper supports were 8.0 mm and 4.0 mm (Figure S1c). The supports had a radius of 0.5 mm at the point of contact with the bone. The load was applied in the anterior to posterior direction so that the anterior side was in compression and the posterior side was in tension. Force and displacement were directly measured by the TestResources system. Stiffness and maximum strength (maximum load) were calculated. Biomechanical properties were normalized by dividing the fractured property by the intact property.

### Histological Evaluation

For histological analysis, femurs were excised, fixed in 10% neutral buffered formalin for at least 24 h, and decalcified in Immunocal decalcifying agent (Decal Chemical Corporation, Tallman, NY) at room temperature for 24 h. The samples were embedded in paraffin, sectioned (5 µm), and stained with hematoxylin and eosin (H&E). Immunohistochemistry was performed for proteins in the PI3K/Pten pathway (p-Akt: #3787, 1∶50 dilution in 0.1% BSA, Cell Signaling Technology, Danvers, MA; p-S6, #4857, 1∶75 dilution; Pten, #9559, 1∶200 dilution; see Document S1). Tartrate-resistant acid phosphatase (TRAP) staining for osteoclasts was performed using a leukocyte acid phosphatase kit (#387A, Sigma-Aldrich, St. Louis, MO) and counterstained with hematoxylin.

### Histomorphometry Analysis

Measurements were performed on Goldner’s Trichrome stained sections (three samples per genotype per day) using Bioquant Osteo 2012 imaging software (Bioquant Imaging Corporation, Nashville, TN). Endosteal bone surfaces were measured within the callus on the outside of the cortical bone on both the medial and lateral side or the anterior and posterior side, depending on the plane of section. The measurement area extended 0.6 mm on either side of the fracture site if possible. All measurement data was pooled. Endosteal bone surfaces were traced within the measurement area. The surfaces were characterized as quiescent, osteoblast surface, and erosion surface with or without osteoclast. Osteoclast surface is the sum of erosion surface with osteoclast and osteoclast surface with no erosion. The osteoblast and osteoclast cell numbers were then counted. Periosteal surfaces were excluded from the measurement area.

### Analysis of Protein Expression

For analysis of protein expression, the fracture callus and corresponding segment of the contralateral femur were harvested and the bone marrow was flushed out of the medullary canal with saline. The femurs were homogenized in a Fast Prep-24 tissue and cell homogenizer (MP Biomedicals, Solon, OH) with Lysing Matrix M (Product # 116923050, MP Biomedicals) and one added ¼” ceramic sphere (Product # 116540034, MP Biomedicals) per sample. The samples were in a p300 lysis buffer [28] supplemented with 0.5 mM sodium orthovanadate. The Fast Prep-24 was run at 6.0 m/s for 40 seconds for four cycles with five minutes in between cycles. The protein lysate was centrifuged at 16,000 g at 4°C for 10 minutes and stored at –80°C. Forty micrograms of lysate were separated on SDS/10% polyacrylamide gels and transferred to nitrocellulose membranes. After probing with primary antibodies (p-Akt, Pten and β-actin at 1∶1000, Product # 4058, 9559 and 4970, respectively, Cell Signaling, Danvers, MA), the membranes were incubated with horseradish peroxidase-linked secondary donkey, anti-rabbit antibodies (Product # 2313, Santa Cruz Biotechnology, Inc., Santa Cruz, CA), and bound antibodies were visualized using Amersham Hyperfilm (Product # 28-9068-35, GE Healthcare, Piscataway, NJ).

Three western blots were scanned in grayscale using a Scanjet G4050 scanner (Hewlett-Packard, Palo Alto, CA) at 1200 dpi. The scans were imported into ImageJ (version 1.46) and the color was inverted so the bands were light on a dark background. A uniform box was drawn around each band and the mean intensity was measured. The intensity of the blot background was determined from the average of three measurements with the same uniform box size. The background was subtracted from the p-Akt, Pten and β-actin bands. The resulting intensity of the p-Akt and Pten bands each were normalized to the intensity of the β-actin band. One common sample was run on each western blot as a standard control. For each blot, the intensity ratio of Pten to β-actin was found for this standard, and all other bands on that western blot were normalized to the intensity ratio of the standard so that band intensity ratios could be compared across separate western blots.

### Statistical Analyses

The effects of genotype and time after fracture were assessed statistically. Since some measures did not satisfy the student’s *t*-test assumptions of normal distribution and equal variance, non-parametric Mann-Whitney U tests were performed on all measures (PASW Statistics 18, SPSS, Inc., Chicago, IL). All analyses were carried out with *p*<0.05 considered significant. Average and standard deviation are reported.

## Results

**Figure 1:**
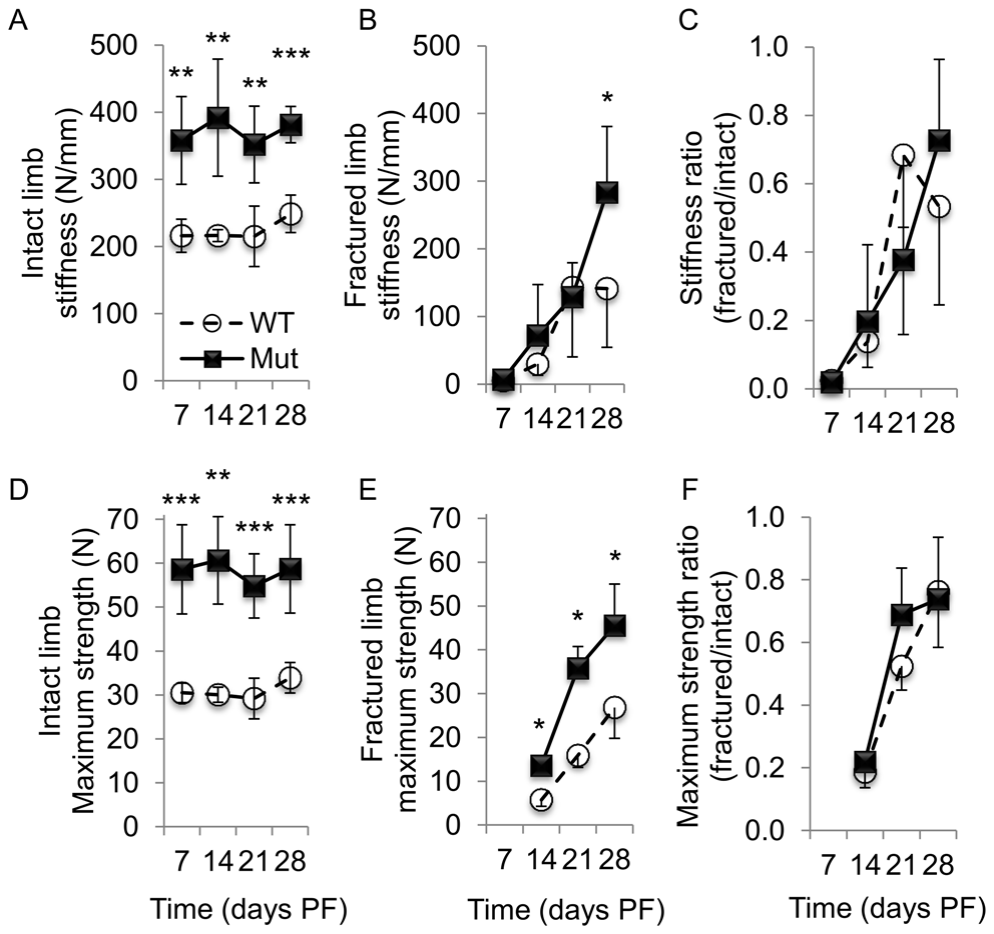
Fracture femoral (A) stiffness and (B) maximum strength. Pten mutants had significantly higher stiffness at 28 d PF and maximum strength at 14, 21, and 28 d PF in fractured femurs. The stiffness and strength increased for both the wild-type and mutant groups during the healing process. (*p<0.05, **p<0.01, and ***p<0.001 WT to Mut at the time point.).

We assessed the stiffness and maximum strength in *Ocn-cre^tg/+;^Pten^flox/flox^* (Pten mutant) and wild-type femurs intact and during the process of fracture repair (Figure 1). Intact contralateral bones from the mutant mice had significantly greater stiffness and maximum load at failure (*p*<0.01 or *p*<0.001) than those of the wild-type animals at each time point (Figure 1a, d). As expected, the stiffness and strength increased throughout healing in both wild-type and mutant animals (Figure 1b, e). Relative to the wild-type, Pten mutants had significantly higher stiffness at 28 days PF and significantly higher maximum strength at 14, 21 and 28 days PF in the fractured femurs (Figure 1b, e). At day 7 PF for both the wild-type and mutant groups, the fractured bone deformed until it was in contact with the lateral sides of the top supports. This changed the distribution of the force at high deformations, and the maximum strength was not applied in the same manner as in the intact loading and other fractured-bone time points. Thus, the maximum strength for day 7 PF of the fractured bones was not included in this analysis. In the wild-type animals, the stiffness at day 28 PF was significantly greater than at days 7 and 14, and the maximum strength at day 28 PF was significantly greater than at days 14 and 21. In Pten mutants, the stiffness at day 28 PF was significantly greater than at days 7, 14, and 21, and the maximum strength at day 28 PF was significantly greater than at day 14.

At all time points, the ratio of the biomechanical properties (stiffness and maximum strength) of the fractured to intact limb was not significantly different between the mutant and wild-type groups (Figure 1c, f). At day 28 PF, the wild-type stiffness and maximum strength were 57% (Figure 1c) and 79% (Figure 1f) of the intact bone, respectively, and the mutant stiffness and force returned to 74% (Figure 1c) and 78% (Figure 1f), respectively, of the values of intact bone. By day 21 PF, the maximum strength for the mutant exceeded that for the wild-type intact bone by 23% (35.8 N for mutant, Figure 1e vs. 29.2 N for wild-type, Figure 1d), and at day 28 PF that difference had increased to 34% (45.5 N for mutant, Figure 1e vs. 33.9 N for wild-type, Figure 1d).

**Figure 2:**
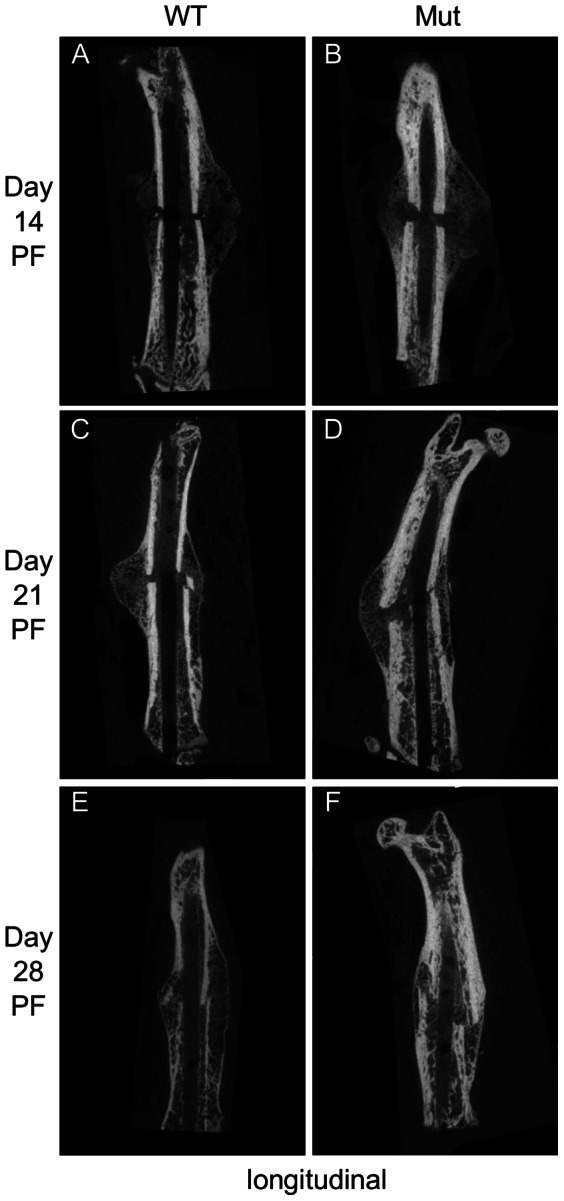
Representative longitudinal µCT sections of the fracture. The Pten mutants had more bone formation at the proximal and distal ends of the fracture callus at each time point.

**Figure 3:**
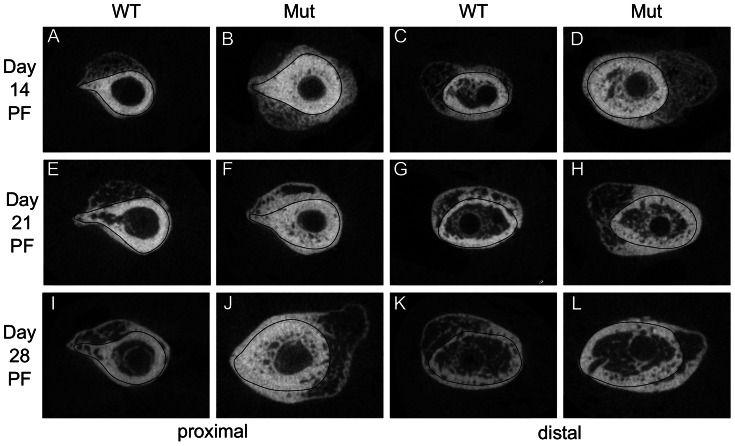
Representative µCT cross sections of bone formation from the periosteum. Sections were taken an average of 2.7 mm away from the fracture. A black line was drawn around the existing bone to indicate the transition between it and newly formed bone. The Pten mutants had more ossification, especially around the existing bone, at each time point.

**Figure 4:**
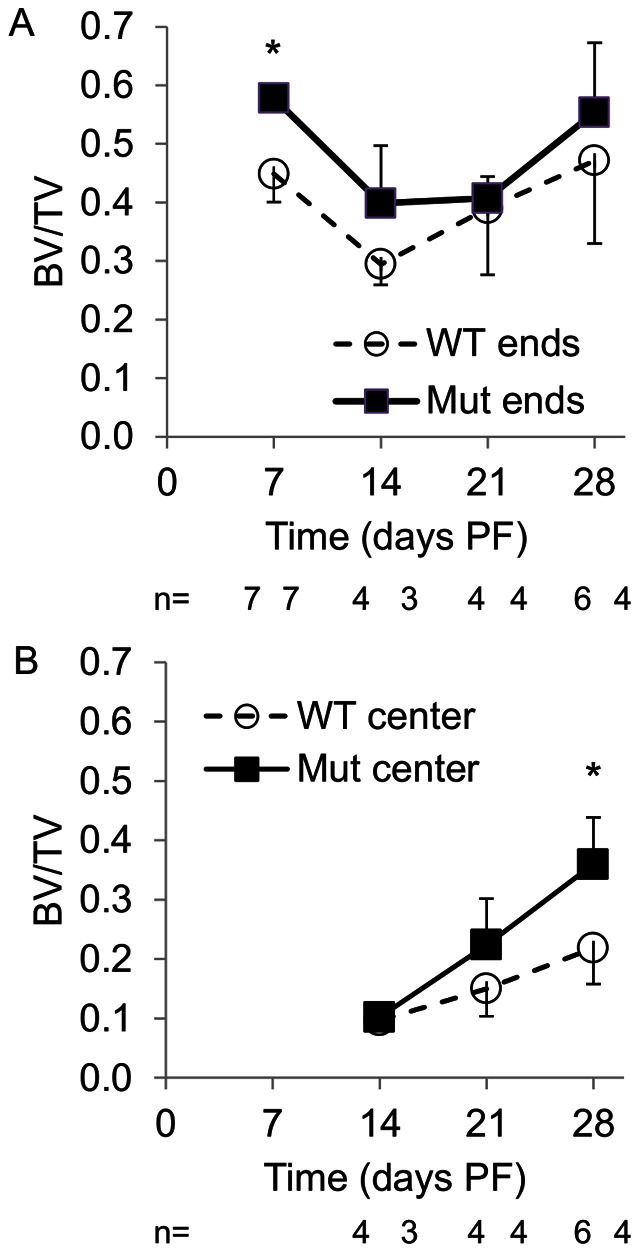
BV/TV µCT callus analysis: (A) BV/TV in proximal and distal callus ends, (B) BV/TV in center of callus. Pten mutants had higher BV/TV at each time point in ends and center. (*p<0.05 WT to Mut at the time point.).

To gain further insight into how Pten deficiency in osteoblasts affects fracture healing, we examined µCT scans (representative longitudinal images are shown in Figure 2 and cross-sectional images in Figure 3). Images were taken an average of 2.7 mm from the fracture to compare the intramembranous ossification arising from the periosteum. Pten mutants had increased ossification in the region away from the fracture (a significant difference at day 7; Figure 4a), where osteogenesis is likely due to intramembranous ossification [4]. At day 21 PF, the intramembranous bone had already grown so dense that it is difficult to distinguish where the existing bone ends and the newly formed bone begins without multiple cross sections (Figures 2 and 3).

**Figure 5:**
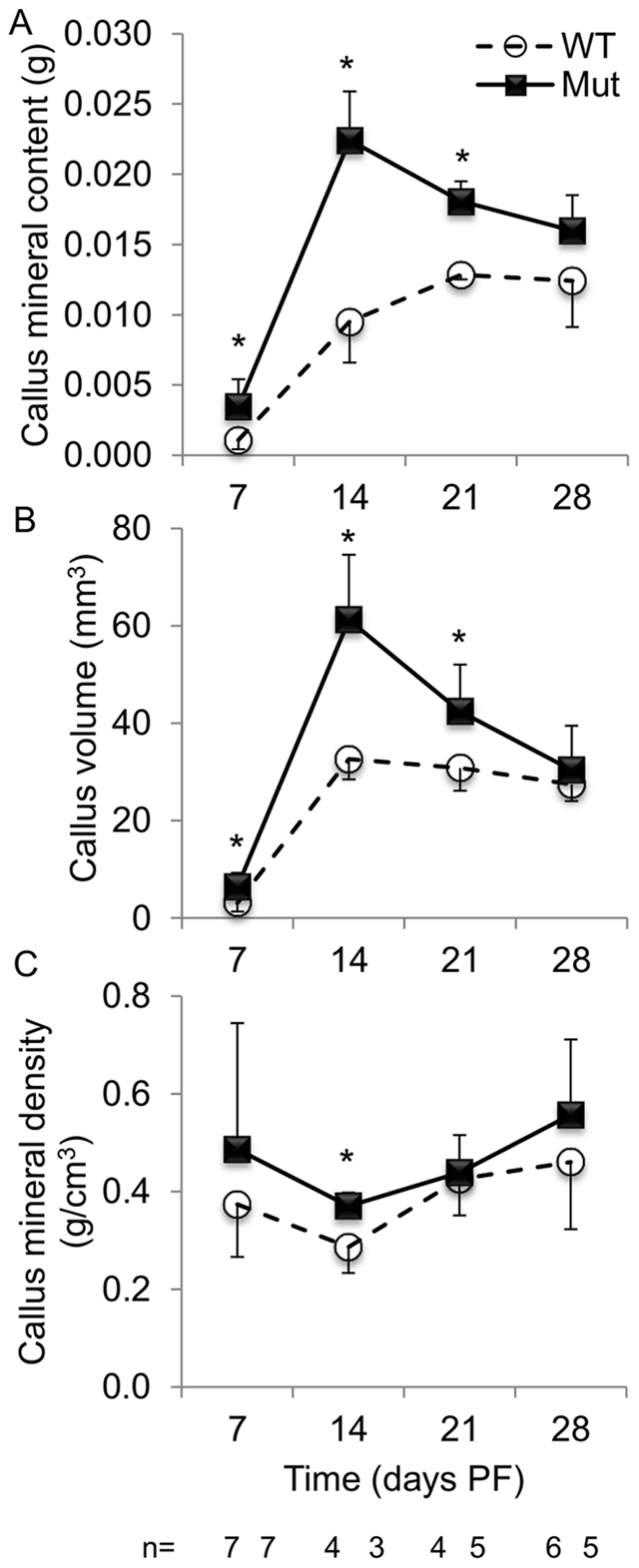
µCT callus analysis: (A) callus mineral content, (B) callus volume, (C) callus mineral density. Pten mutants had more callus mineral content, volume and density at each time point. (*p<0.05 WT to Mut at the time point.).

We calculated BV/TV (Figure 4), callus mineral content, volume, and density (Figure 5) from the µCT data. At day 7 PF, the mutants had significantly higher BV/TV in the callus ends and at day 28 PF the mutants had significantly higher BV/TV in the central callus. At days 7, 14, and 21 PF, the mutants had significantly more mineral content and volume than the wild-type animals. The mutants had a denser callus at each time point, but the difference was only significant on day 14 PF.

**Figure 6:**
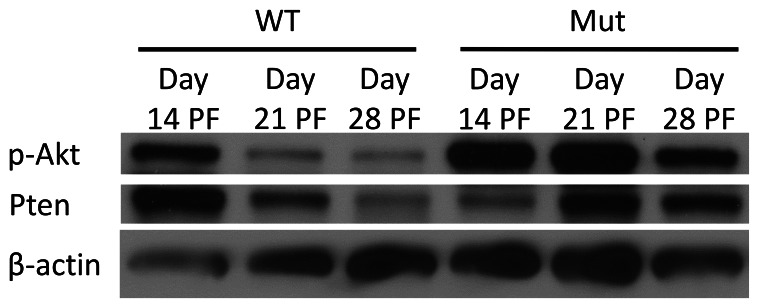
p-Akt and Pten protein expression during healing. Pten mutants had increased p-Akt expression at days 21 and 28 PF.

We performed western blotting to analyze the protein expression in the fracture calluses throughout the healing process. The expression of phosphorylated Akt (p-Akt) was increased in the mutants at days 14, 21, and 28 days PF but the difference was not significant (Figure 6; Figure S2).

**Figure 7:**
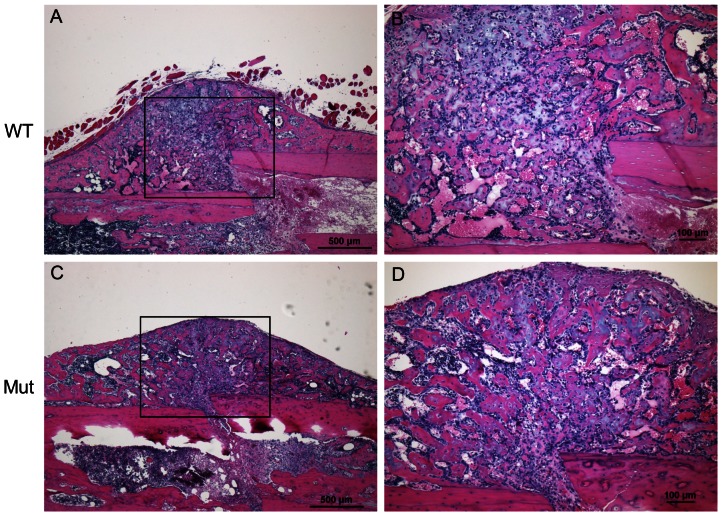
H&E of fracture calluses at day 14 PF. (A) 4× magnification of wild-type callus; (B) 10× magnification of the box from (A); (C) 4× magnification of Pten mutant callus; and (D) 10× magnification of box from (C). Woven bone was spread throughout the callus and was surrounded hypertrophic chondrocytes in both cases.

We performed histological analysis on the fracture calluses. At day 7 PF, each callus was composed of mainly fibroblast cells and chondrocytes (Figure S3). By day 14 PF, woven bone had replaced some of the chondrocytes and was apparent throughout the callus (Figure 7). There were regions where the woven bone surrounds hypertrophic chondrocytes, which demonstrated that the bone was undergoing endochondral ossification. By day 21 PF, bone had replaced most of the cartilage matrix (Figures S4 and S5).

**Figure 8:**
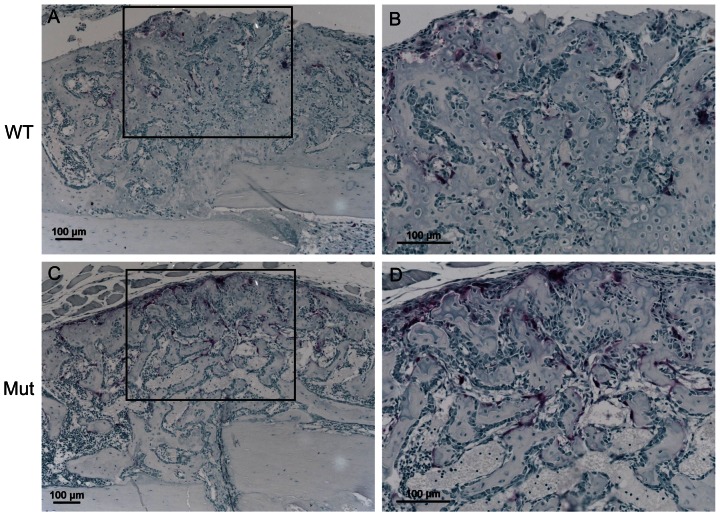
TRAP stain of fracture calluses at day 14 PF. (A) 10× magnification of wild-type callus; (B) 20× magnification of box from (A); (C) 10× magnification of Pten mutant callus; (D) 20× magnification of box from (C). TRAP staining was more intense in the mutant group.

We performed immunohistochemistry analysis on the fracture calluses. The Pten IHC staining was similar between the wild-type and mutant groups in the callus at day 7 PF but was more intense in the mutant group in the bone lining cells at days 14, 21 and 28 PF (Figures S6, S7, S8, S9). The expression of phosphorylated Akt (p-Akt) was observed in bone lining cells surrounding the newly formed bone and was similar in the wild-type and mutant groups at day 14 PF (Figure S10) and 21 PF (Figure S11). The expression of phosphorylated ribosomal S6 kinase 1 (p-S6) was observed in bone lining cells surrounding the newly formed bone and was similar in the wild-type and mutant groups at day 14 PF (Figure S12) and 21 PF (Figure S13). TRAP staining was increased in the mutant group at days 14 and 21 (Figures 8 and S14).

## Discussion

Relative to wild-type mice, mice lacking Pten in their osteoblasts had significantly stiffer and stronger intact bones at all time points. This result confirms the previous study in which the bones of the Pten mutants had a higher bone mineral density and more midshaft cortical bone in the femur [17], i.e. bone density and material distribution contribute to biomechanical stiffness [29].

Biomechanical analysis also showed that Pten mutants had significantly stiffer (day 28 PF) and stronger (days 14, 21 and 28 PF) healing bones at the later stages of the healing process. At earlier stages of healing, the increase in newly formed bone at the proximal and distal ends of the callus (Figure 4a) by itself contributed little to the overall biomechanical stiffness and strength of the callus: during biomechanical testing, the callus broke at the weakest point, which was the more central cartilaginous region near the original fracture. That the biomechanical difference between the two groups was not apparent until later in the healing process (days 14, 21 and 28 PF) is expected because the only cells in which Pten is absent express osteocalcin, and those cells do not appear near the fracture site until day 10 or 14 PF. Reporter mice for osterix [20], collagen I alpha I [30], and osteocalcin [30] activity have defined the location of cells from the osteoblastic lineage during fracture repair. Osterix-positive osteoblast precursors entered the callus along with the invading blood vessels [20]. Collagen I alpha I cells were apparent in the callus by day 4 PF; at day 7 PF, osteocalcin-positive cells were present near the bone originating from the periosteum at the proximal and distal ends of the callus but not near the fracture site [30]. As early as day 10 PF, osteocalcin-positive cells were evident in the callus, although they were still less common than the collagen I alpha I-positive cells; the more prevalent collagen I alpha I-positive cells indicate the cells are earlier in the osteoblast differentiation process [30]. At day 14 PF, mature osteoblasts were located near the outer shell of the callus near the fracture site [30] where the addition of bone is the most biomechanically advantageous [29], and throughout the callus [31]. The improved stiffness and maximum strength of fractured bones from the Pten mutants is consistent with the increased BV/TV in the central callus at that time (Figure 4b) and demonstrated improved fracture healing. By day 21 PF, the fracture callus of the Pten mutants was stronger than the wild-type intact bone, which further demonstrated that the inhibition of Pten enhanced fracture healing. Increased ossification at the proximal and distal ends of the callus (Figure 4a) supports the inference that the inhibition of Pten enhanced intramembranous ossification. The ratio of the biomechanical properties in the fractured limb to the intact limb was not significantly different between the wild-type and mutants (Figure 1c, f). This indicates that fracture healing of the bone occurs at the same rate as the growth of the bone in each group, i.e. Pten mutants grow bone better in development (Figure 1a, d) and fracture healing (Figure 1b, e).

The qualitative appearance of the callus did not appear different between the mutant and wild-type groups in the H&E stained images (Figures 7 and S3, S4, S5). The wild-type and mutant groups appeared to heal with similar kinetics in the same manner. The progression of healing in the cartilaginous callus followed the same pattern as that of bone built during normal endochondral ossification. However, at a later time point in fracture healing when mature osteoblasts that produce osteocalcin were present, the Pten mutants had decreased Pten expression (Figures S6, S7, S8, S9), and increased ossification, as shown by the increased thickness of bone at the proximal and distal ends of the callus. This also likely occurred near the fracture site later in the healing and is consistent with the observation that the biomechanical strength and stiffness of the Pten mutants were both improved only at day 28 PF relative to the wild-type intact femur. The Pten mutants also had increased TRAP staining at days 14 and 21 PF (Figures 8 and S14). The mutants also had significantly more osteoclasts, osteoclast surface, and eroded surface in the fracture callus (Table 1) at day 14 PF, which is consistent with our characterization of the mice [17], though these differences were not significant when normalized to bone surface (Table 1). This demonstrates that the mutant bones are not stronger because of decreased osteoclast activity. It is expected that at later time points than those studied here, the difference in the strength and stiffness between the mutants and wild-type would be more pronounced, because the Pten-deficient osteoblasts will continue to provide more mineralization than the wild-type osteoblasts. Protein extracts showed decreased Pten and increased Akt signaling (p-Akt) in the mutants at day 21 PF. The expression of Pten in the mutants could be from cells other than osteoblasts in the fracture callus (e.g., chondrocytes and fibroblasts).

**Table 1:** Callus histomorphometric measures (*p<0.05 WT to Mut at the time point).

		14 days PF		21 days PF	
Measure	Abbreviation	WT	Mut	WT	Mut
Bone surface (mm)	BS	27±8	30±2	28±1	23±2*
Osteoblast surface (mm)	Ob.S	8.7±4.6	9.9±1.2	5.7±2.6	5.9±2.8
Osteoclast surface (mm)	Oc.S	1.6±0.1	2.3±0.3*	2.1±0.4	1.8±0.3
Erosion surface (mm)	ES	1.9±0.2	2.9±0.1*	3.6±0.3	2.9±0.2*
Erosion surface with osteoclast (mm)	ES(Oc+)	1.6±0.1	2.3±0.3*	2.1±0.4	1.8±0.3
Number of osteoblasts	N.Ob	561±277	682±74	402±189	425±198
Number of osteoclasts	N.Oc	53±4	69±4*	63±12	55±12
Osteoblast surface per bone surface (%)	Ob.S/BS	31±7	33±2	20±9	25±11
Osteoclast surface per bone surface (%)	Oc.S/BS	6.2±1.5	7.7±0.8	7.3±1.6	7.7±0.9
Erosion surface per bone surface (%)	ES/BS	7.8±2.8	9.5±1.1	12.8±1.6	12.9±0.8
Erosion surface with osteoclast per bonesurface (%)	ES(Oc+)/BS	6.2±1.5	7.6±0.8	7.0±1.5	7.5±0.8
Number of osteoblasts per bone perimeter(#/mm)	N.Ob/B.Pm	20±4	22±2	14±7	18±8
Number of osteoclasts per bone perimeter(#/mm)	N.Oc/B.Pm	2.1±0.4	2.3±0.2	2.2±0.4	2.4±0.3

This study used a computational measure of the three-dimensional callus characteristics to assess fracture healing. Previous studies have used select slices in CT scan analysis [26], [32]. Our method demonstrated an increased callus mineral content and volume in the Pten mutant mice relative to the wild-type. The improved callus mineral content and mineralized callus volume indicated that mice lacking Pten in osteoblasts healed more robustly than the wild-type mice. The observed increase in callus size and callus mineral content is expected to increase callus strength. These results indicate that inhibition of Pten could improve fracture healing and could be a candidate treatment for non-union.

Future work should address whether Pten can be inhibited locally and can improve the growth of bone after fracture. Despite the fact that Pten is a known tumor suppressor [15], transient inhibition of Pten is being evaluated to treat other conditions. For example, there has already been work in developing agents that inhibit the activity of Pten to treat diabetes [33], provide cardiac protection against ischemia/reperfusion injury [34], reduce the severity of acute lung injury [35], and accelerate wound closure [36]. Some of these agents are commercially available, and it is crucial to note that such agents have already been used in mouse models with no evidence of deleterious effects. Given that that the half-life of Pten protein is 48–72 hours [37] and the half-life of this inhibitor is also relatively short [35], a strategy of transient inhibition should be practical. Such a strategy would further reduce the chances of any negative effects. The results of this study indicate that the inhibition of Pten can improve fracture healing, and that the local or short-term transient use of these Pten-inhibiting agents has potential clinical applications to enhance fracture healing.

## Supporting Information

Figure S1 **Schematics of µCT scanning and mechanical testing setup.** (A) Representative screenshots of callus cropped fractured bone (darker gray) and intact bone (lighter gray) masks from one mouse 14 d PF. (B) Schematic demonstrating how the callus volume and mineral content was calculated from Mimics. Fractured bone and callus (darker gray) minus the bone (lighter gray) equals the callus (darkest gray). The horizontal lines indicate where the masks were cropped. (C) Mechanical testing setup for fractured bone and callus (darker gray) and intact bone (lighter gray).
(TIF)

Figure S2 **Quantitation of western blots.** (A) Mutant animals trended to have more normalized p-Akt than wildtype animals. (B) Mutant animals trended to have less normalized Pten than wildtype animals.
(TIF)

Figure S3 **H&E of fracture calluses at day 7 PF.** (A) 4× magnification of wild-type callus; (B) 10× magnification of the box from (A); (C) 4× magnification of Pten mutant callus; and (D) 10× magnification of the box from (C). The fracture callus consisted of mostly fibroblast cells and chondrocytes in each case.
(TIF)

Figure S4 **H&E of fracture calluses at day 21 PF.** (A) 4× magnification of wild-type callus; (B) 10× magnification of box from (A); (C) 4× magnification of Pten mutant callus; and (D) 10× magnification of box from (C). Woven bone had replaced the cartilage matrix in each case.
(TIF)

Figure S5 **H&E of fracture calluses at day 28 PF.** (A) 4× magnification of wild-type callus; (B) 10× magnification of box from (A); (C) 4× magnification of Pten mutant callus; and (D) 10× magnification of box from (C). The callus consists of mostly woven bone in each case.
(TIF)

Figure S6 **Pten IHC of fracture calluses at day 7 PF.** (A) 10× magnification of wild-type callus; (B) 20× magnification of box from (A); (C) 10× magnification of Pten mutant callus; (D) 20× magnification of box from (C). Pten was expressed at a similar level in each case.
(TIF)

Figure S7 **Pten IHC of fracture calluses at day 14 PF.** (A) 10× magnification of wild-type callus; (B) 20× magnification of box from (A); (C) 10× magnification of Pten mutant callus; (D) 20× magnification of box from (C). Pten was expressed at a higher level in the bone lining cells in the wildtype animals.
(TIF)

Figure S8 **Pten IHC of fracture calluses at day 21 PF.** (A) 10× magnification of wild-type callus; (B) 20× magnification of box from (A); (C) 10× magnification of Pten mutant callus; (D) 20× magnification of box from (C). Pten was expressed at a higher level in the bone lining cells in the wildtype animals.
(TIF)

Figure S9 **Pten IHC of fracture calluses at day 28 PF.** (A) 10× magnification of wild-type callus; (B) 20× magnification of box from (A); (C) 10× magnification of Pten mutant callus; (D) 20× magnification of box from (C). Pten was expressed at a higher level in the bone lining cells in the wildtype animals.
(TIF)

Figure S10 **p-Akt IHC of fracture calluses at day 14 PF.** (A) 10× magnification of wild-type callus; (B) 20× magnification of box from (A); (C) 10× magnification of Pten mutant callus; (D) 20× magnification of box from (C). p-Akt was expressed at a similar level in each case.
(TIF)

Figure S11 **p-Akt IHC of fracture calluses at day 21 PF.** (A) 10× magnification of wild-type callus; (B) 20× magnification of box from (A); (C) 10× magnification of Pten mutant callus; (D) 20× magnification of box from (C). p-Akt was expressed at a similar level in each case.
(TIF)

Figure S12 **p-S6 IHC of fracture calluses at day 14 PF(A) 10**× **magnification of wild-type callus; (B) 20**× **magnification of box from (A); (C) 10**× **magnification of Pten mutant callus; (D) 20**× **magnification of box from (C).** p-S6 was expressed at a similar level in each case.
(TIF)

Figure S13 **p-S6 IHC of fracture calluses at day 21 PF.** (A) 10× magnification of wild-type callus; (B) 20× magnification of box from (A); (C) 10× magnification of Pten mutant callus; (D) 20× magnification of box from (C). p-S6 was expressed at a similar level in each case.
(TIF)

Figure S14 **TRAP stain of fracture calluses at day 21 PF.** (A) 10× magnification of wild-type callus; (B) 20× magnification of box from (A); (C) 10× magnification of Pten mutant callus; (D) 20× magnification of box from (C). TRAP staining was more intense in the mutant group.
(TIF)

Document S1 **Supplemental methods.**
(DOCX)

## Acknowledgments

The authors thank the members of the Mason and Williams labs at VARI for their assistance; David Nadziejka for assistance preparing the manuscript; DJ Scholten for assisting on surgeries and fractures; Lisa Turner and the VARI histology core for their histology work; Michael Starbuck in the Rolanette and Berdon Lawrence Bone Disease Program of Texas bone histomorphometry lab for his help with the histomorphometry analysis; VARI’s vivarium staff for outstanding animal husbandry; and Luci Korpi and the Grand Rapids Area Pre-College Engineering Program for helping with this research.
