# Supplementary figures and images for: Mice Lacking Pten in Osteoblasts Have Improved Intramembranous and Late Endochondral Fracture Healing
Source: PLoS One. 2013 May 13;8(5):e63857. doi: 10.1371/journal.pone.0063857 (PMC3652860; doi:10.1371/journal.pone.0063857)

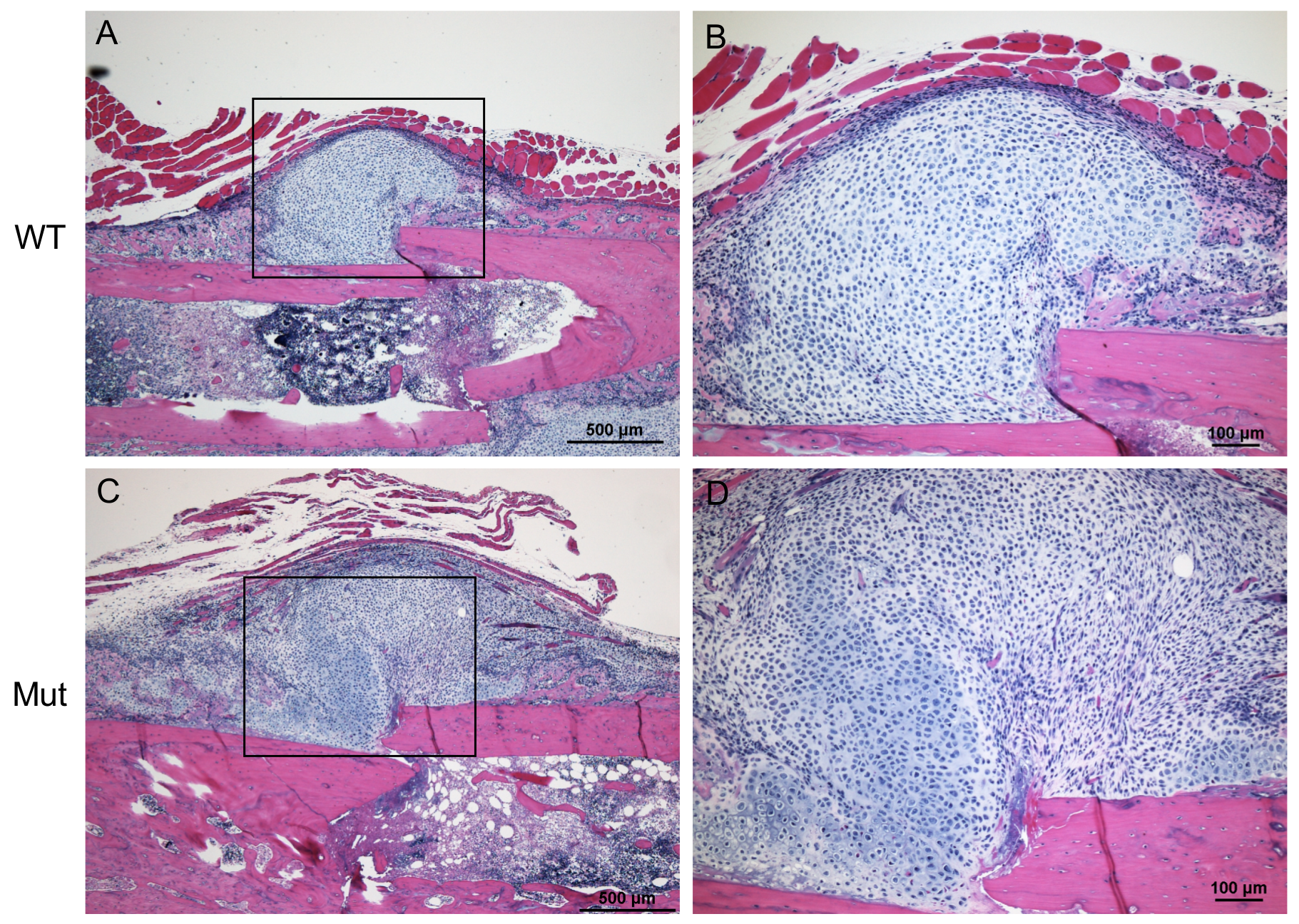

Supplement: Figure S3 — H&E of fracture calluses at day 7 PF. (A) 4× magnification of wild-type callus; (B) 10× magnification of the box from (A); (C) 4× magnification of Pten mutant callus; and (D) 10× magnification of the box from (C). The fracture callus consisted of mostly fibroblast cells and chondrocytes in each case. (TIF) [file pone.0063857.s003.tif]

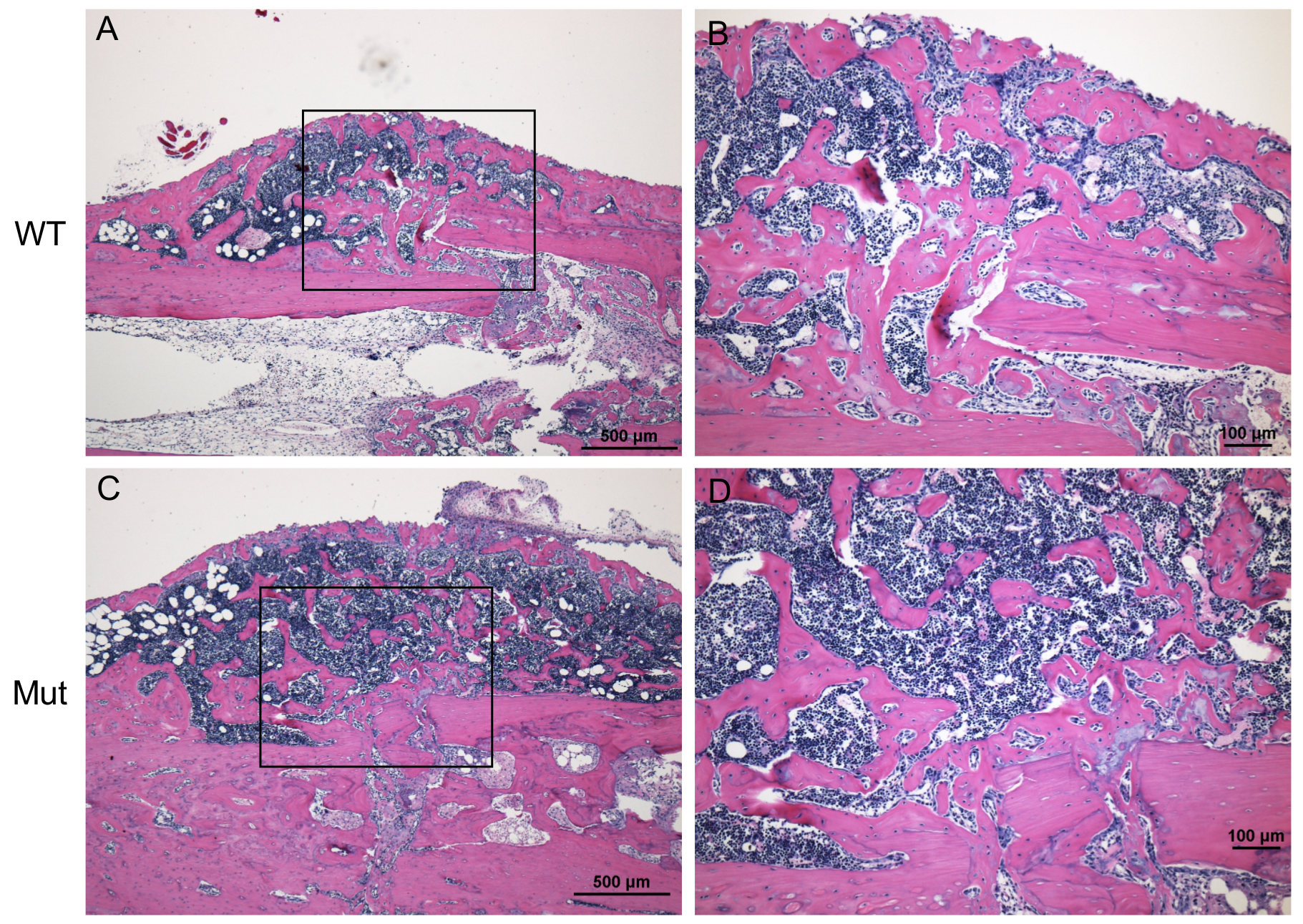

Supplement: Figure S4 — H&E of fracture calluses at day 21 PF. (A) 4× magnification of wild-type callus; (B) 10× magnification of box from (A); (C) 4× magnification of Pten mutant callus; and (D) 10× magnification of box from (C). Woven bone had replaced the cartilage matrix in each case. (TIF) [file pone.0063857.s004.tif]

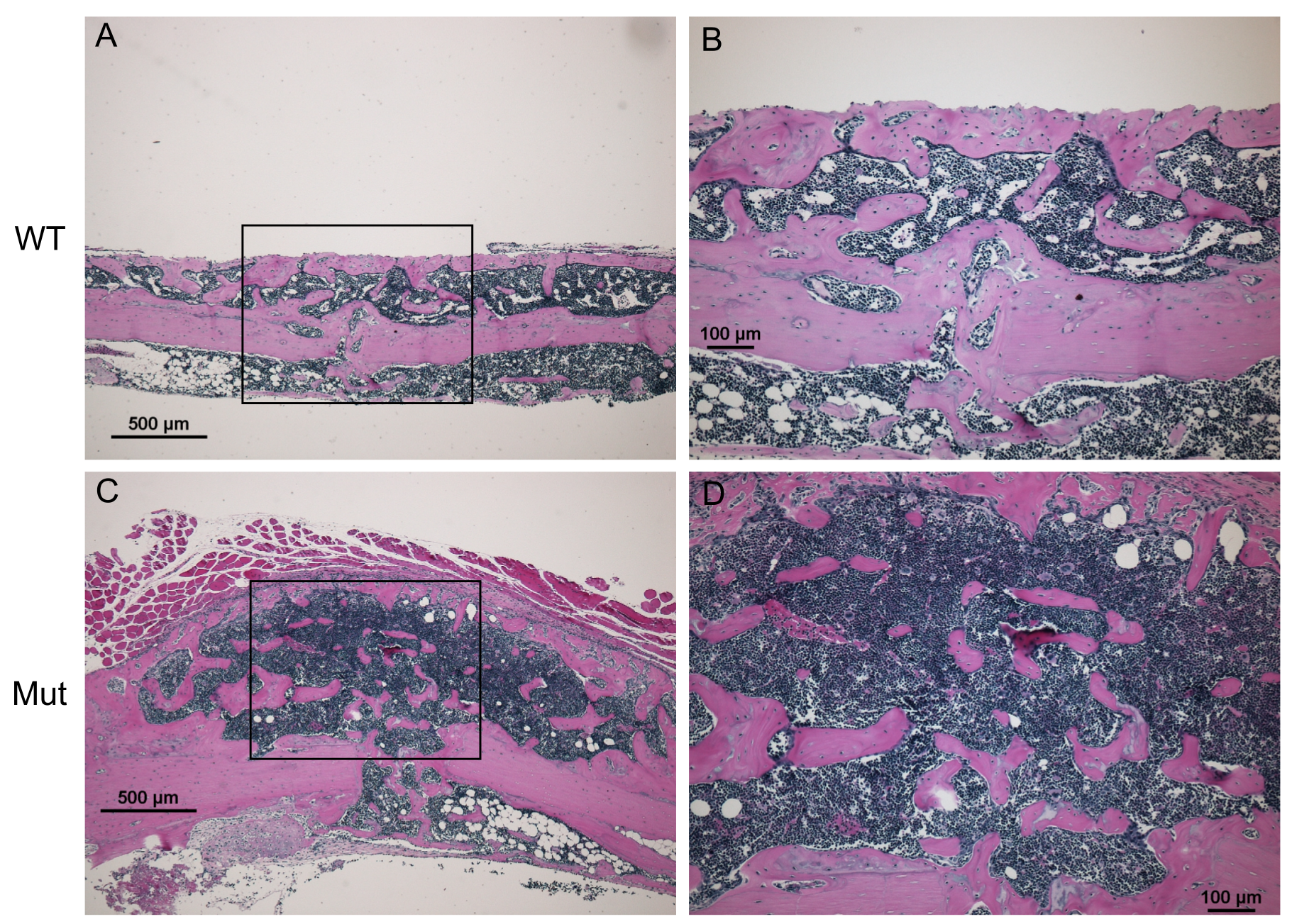

Supplement: Figure S5 — H&E of fracture calluses at day 28 PF. (A) 4× magnification of wild-type callus; (B) 10× magnification of box from (A); (C) 4× magnification of Pten mutant callus; and (D) 10× magnification of box from (C). The callus consists of mostly woven bone in each case. (TIF) [file pone.0063857.s005.tif]

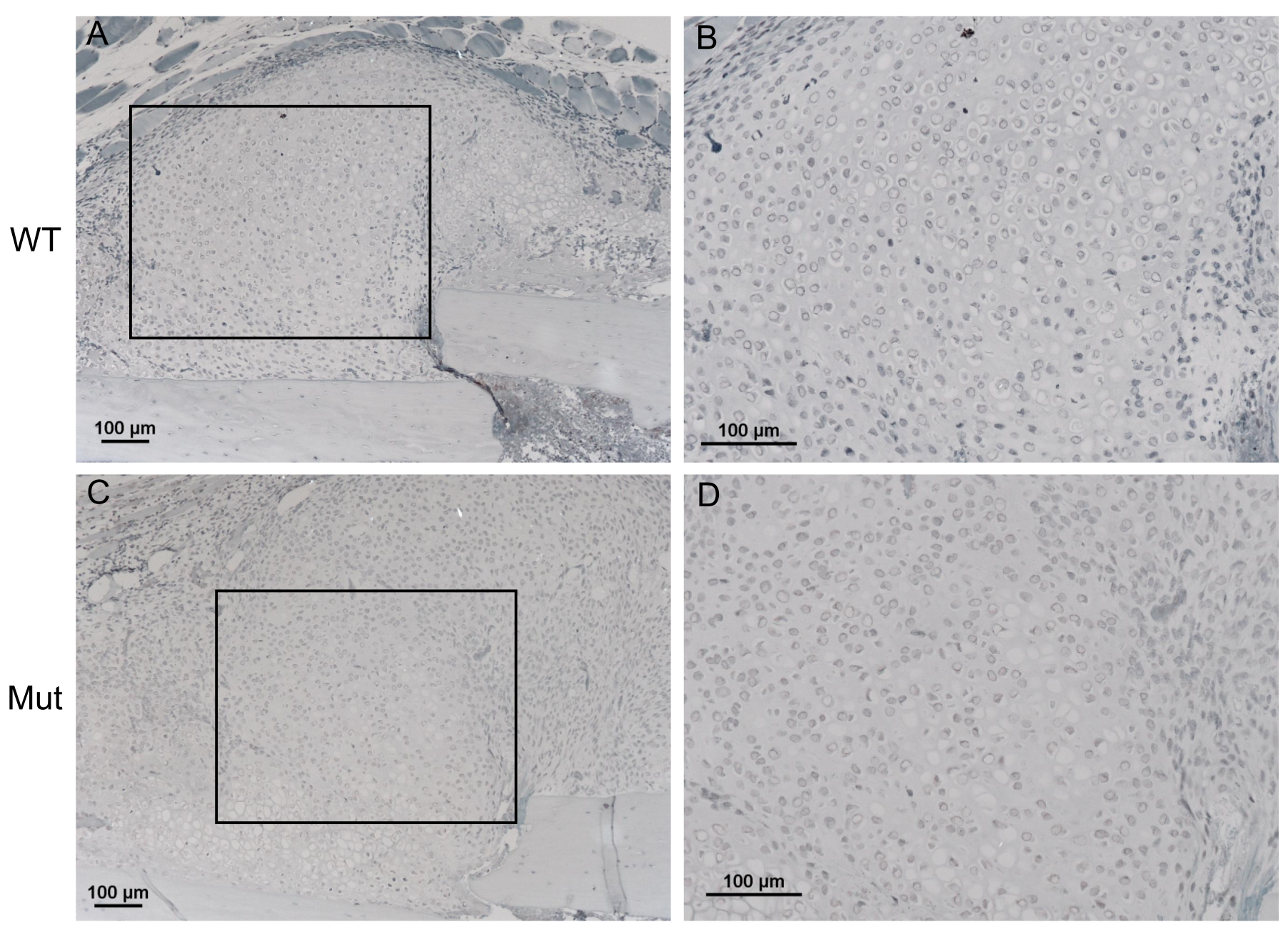

Supplement: Figure S6 — Pten IHC of fracture calluses at day 7 PF. (A) 10× magnification of wild-type callus; (B) 20× magnification of box from (A); (C) 10× magnification of Pten mutant callus; (D) 20× magnification of box from (C). Pten was expressed at a similar level in each case. (TIF) [file pone.0063857.s006.tif]

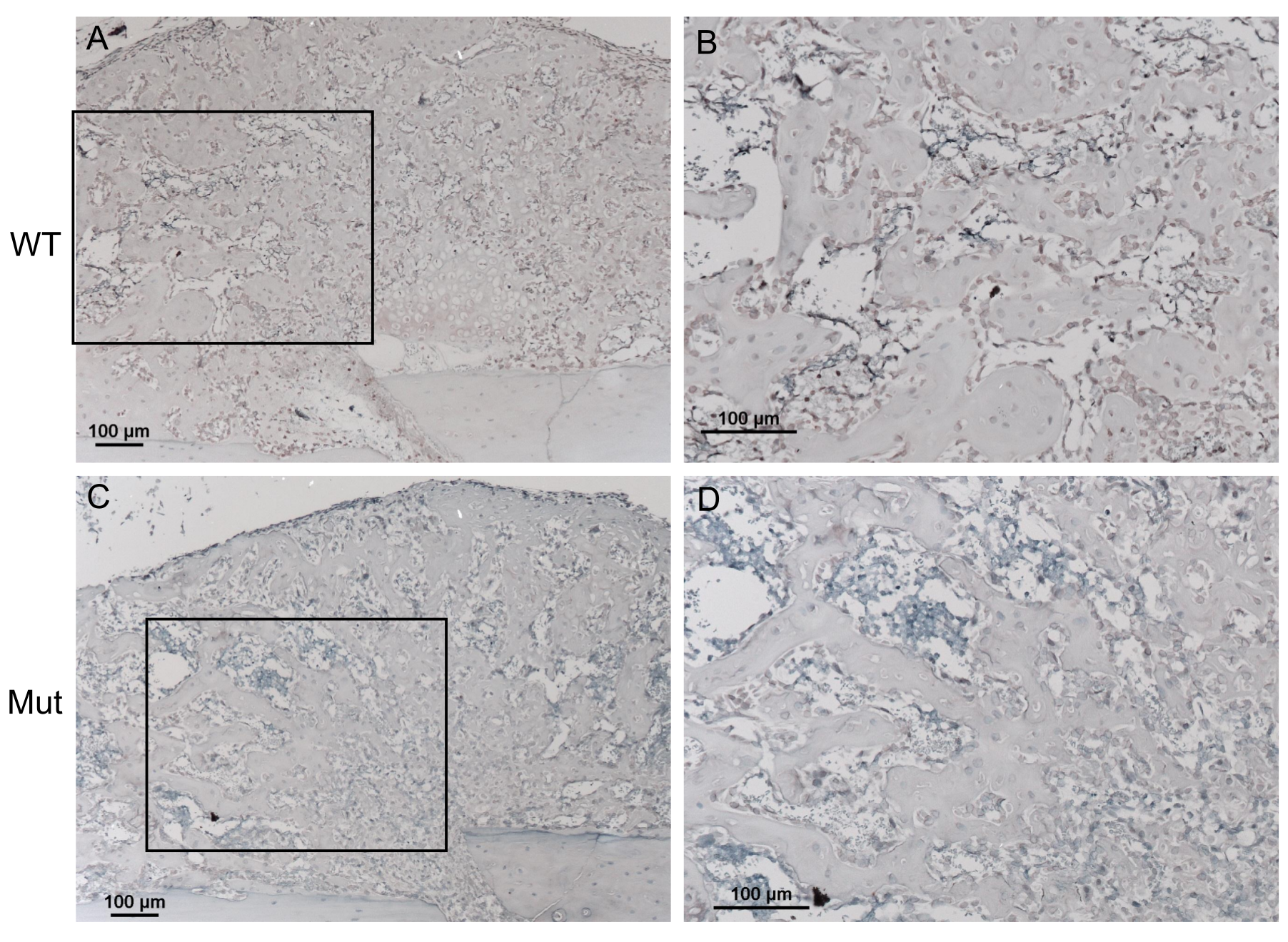

Supplement: Figure S7 — Pten IHC of fracture calluses at day 14 PF. (A) 10× magnification of wild-type callus; (B) 20× magnification of box from (A); (C) 10× magnification of Pten mutant callus; (D) 20× magnification of box from (C). Pten was expressed at a higher level in the bone lining cells in the wildtype animals. (TIF) [file pone.0063857.s007.tif]

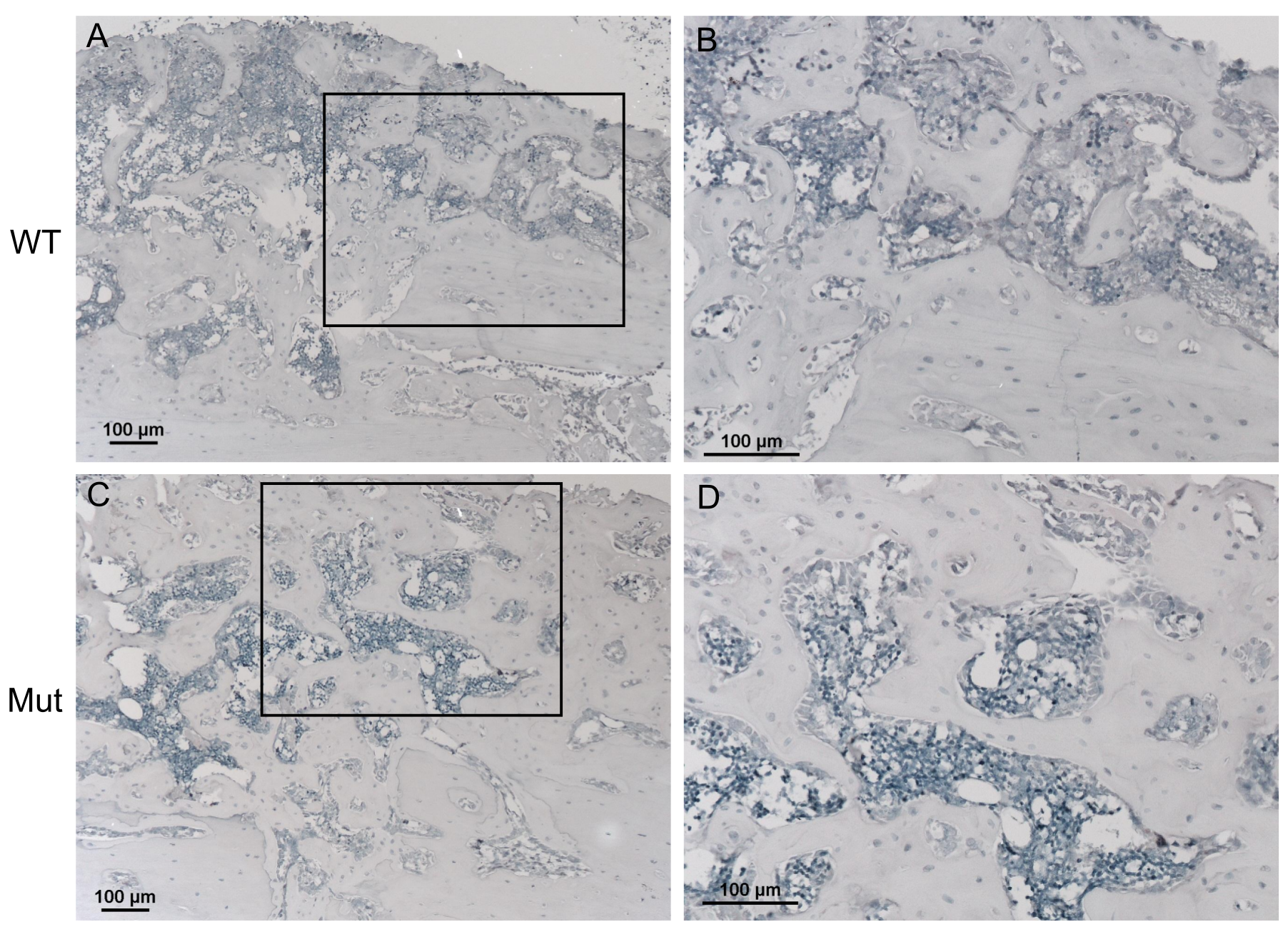

Supplement: Figure S8 — Pten IHC of fracture calluses at day 21 PF. (A) 10× magnification of wild-type callus; (B) 20× magnification of box from (A); (C) 10× magnification of Pten mutant callus; (D) 20× magnification of box from (C). Pten was expressed at a higher level in the bone lining cells in the wildtype animals. (TIF) [file pone.0063857.s008.tif]

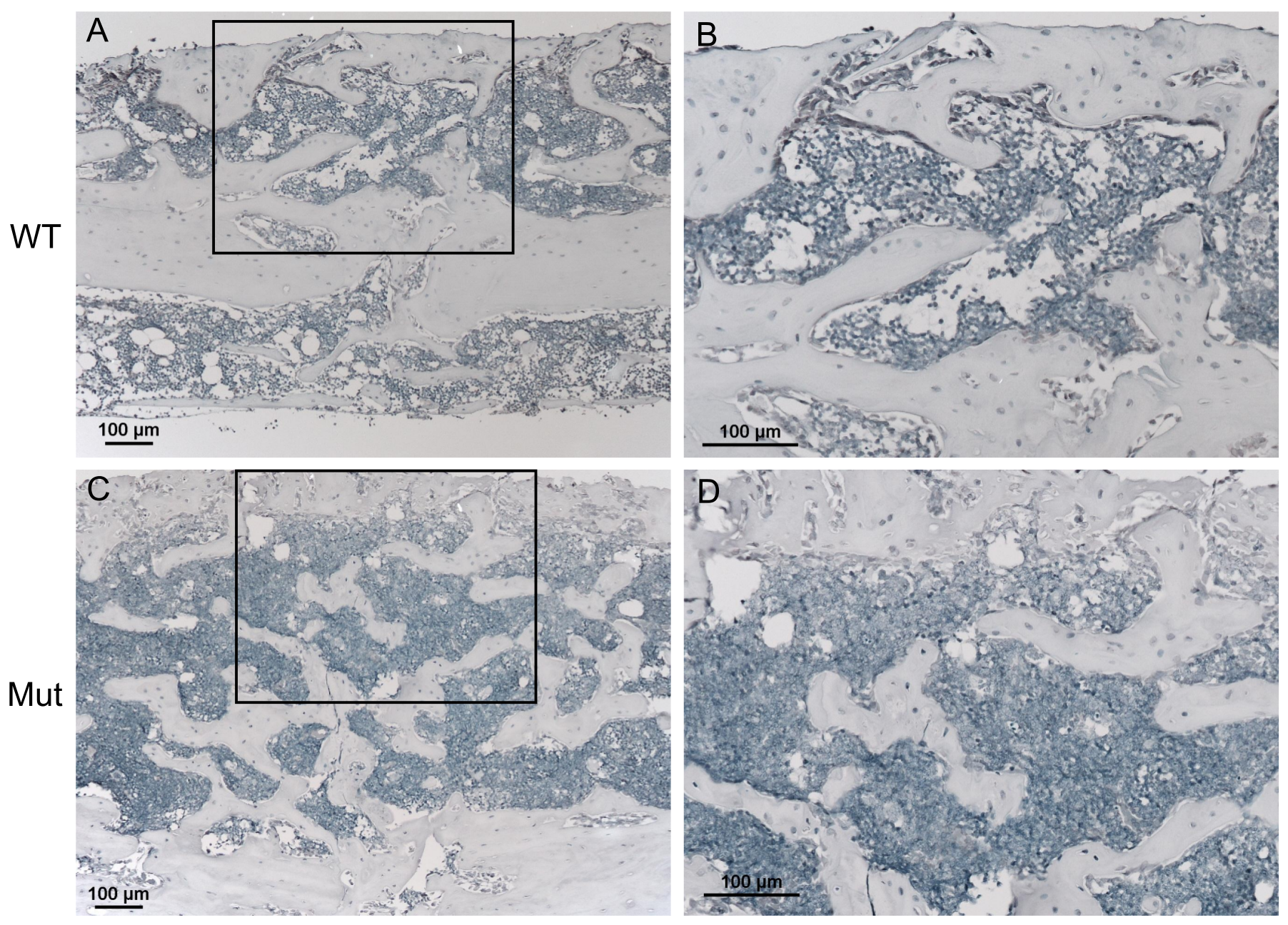

Supplement: Figure S9 — Pten IHC of fracture calluses at day 28 PF. (A) 10× magnification of wild-type callus; (B) 20× magnification of box from (A); (C) 10× magnification of Pten mutant callus; (D) 20× magnification of box from (C). Pten was expressed at a higher level in the bone lining cells in the wildtype animals. (TIF) [file pone.0063857.s009.tif]

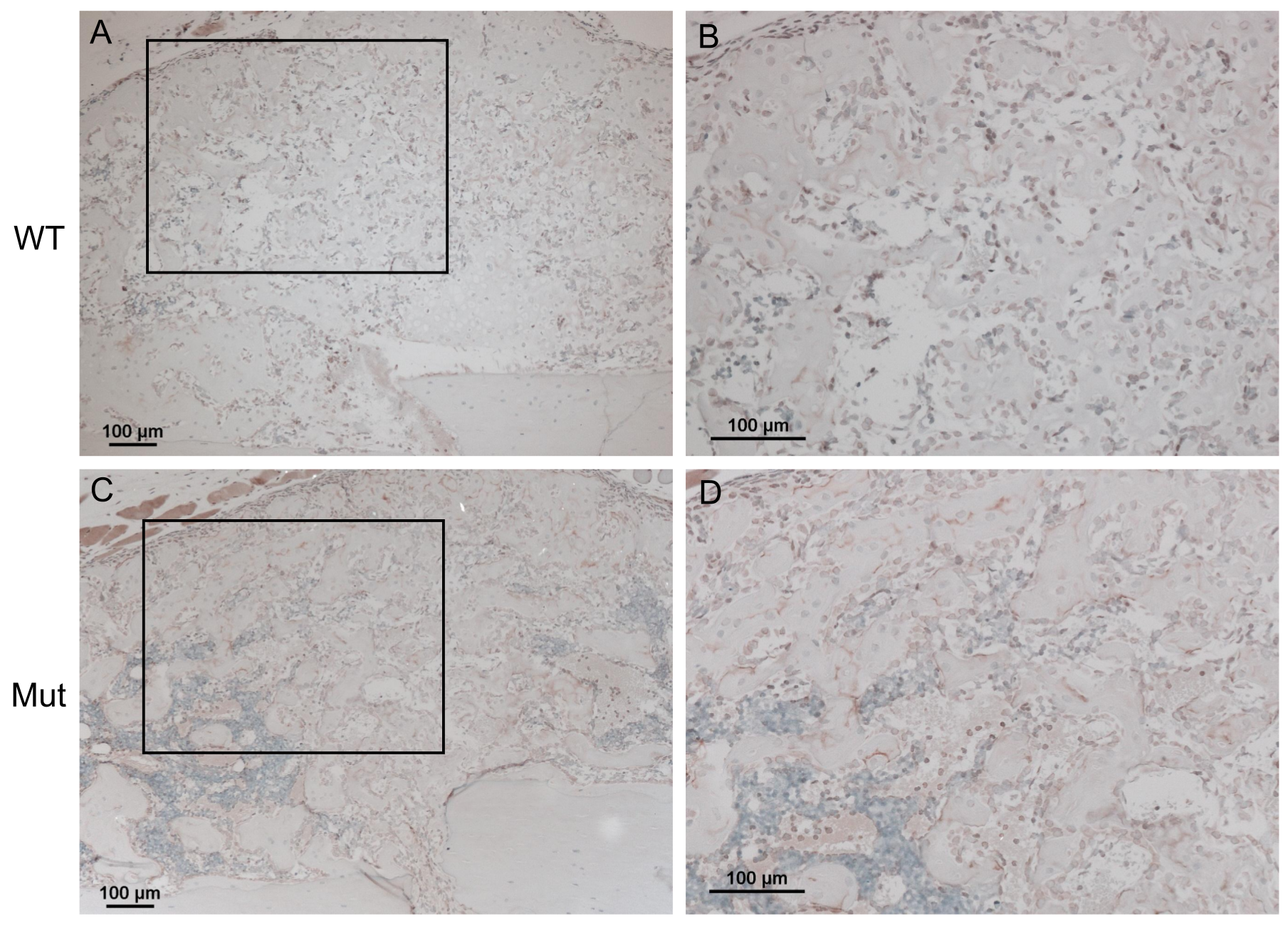

Supplement: Figure S10 — p-Akt IHC of fracture calluses at day 14 PF. (A) 10× magnification of wild-type callus; (B) 20× magnification of box from (A); (C) 10× magnification of Pten mutant callus; (D) 20× magnification of box from (C). p-Akt was expressed at a similar level in each case. (TIF) [file pone.0063857.s010.tif]

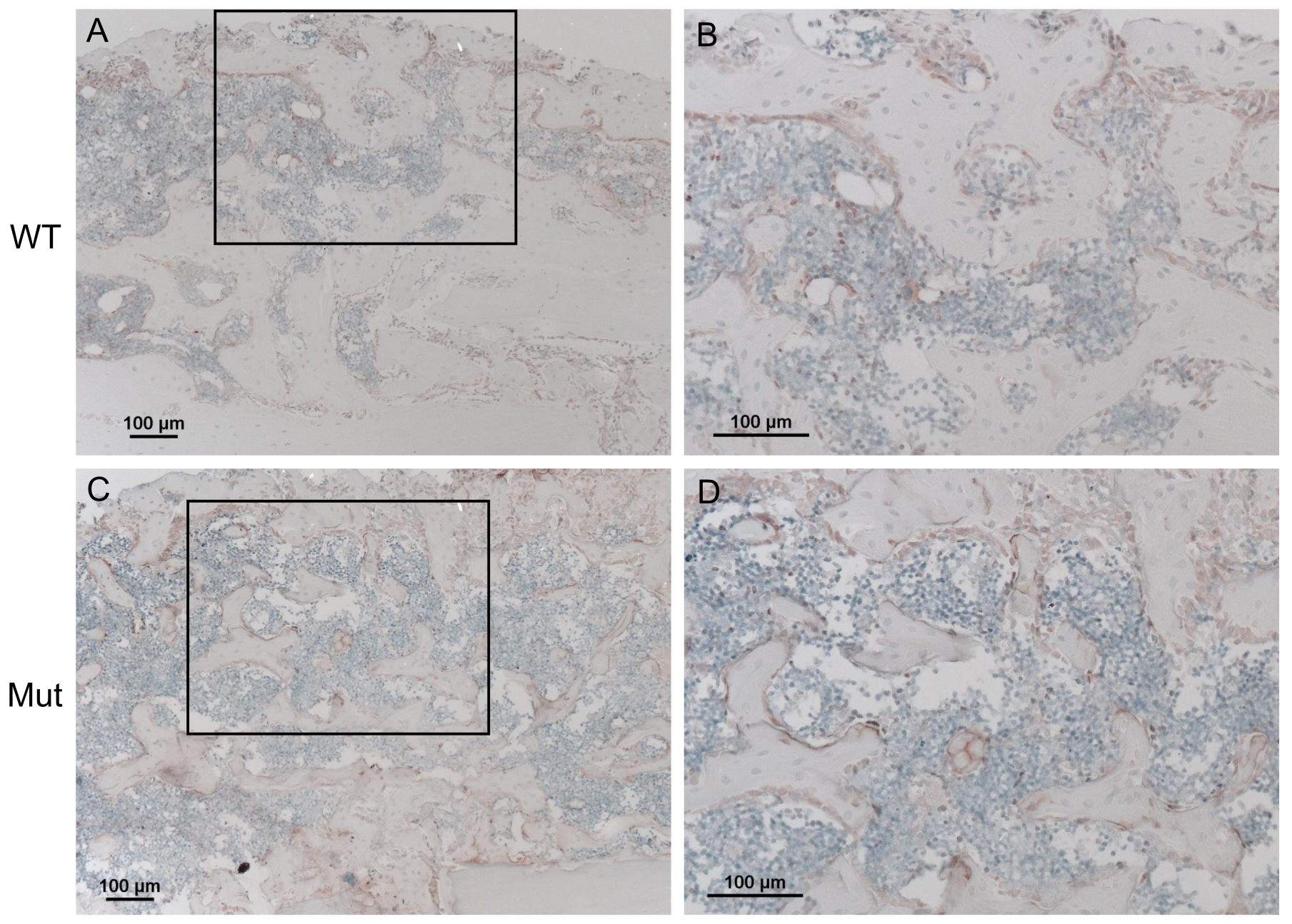

Supplement: Figure S11 — p-Akt IHC of fracture calluses at day 21 PF. (A) 10× magnification of wild-type callus; (B) 20× magnification of box from (A); (C) 10× magnification of Pten mutant callus; (D) 20× magnification of box from (C). p-Akt was expressed at a similar level in each case. (TIF) [file pone.0063857.s011.tif]

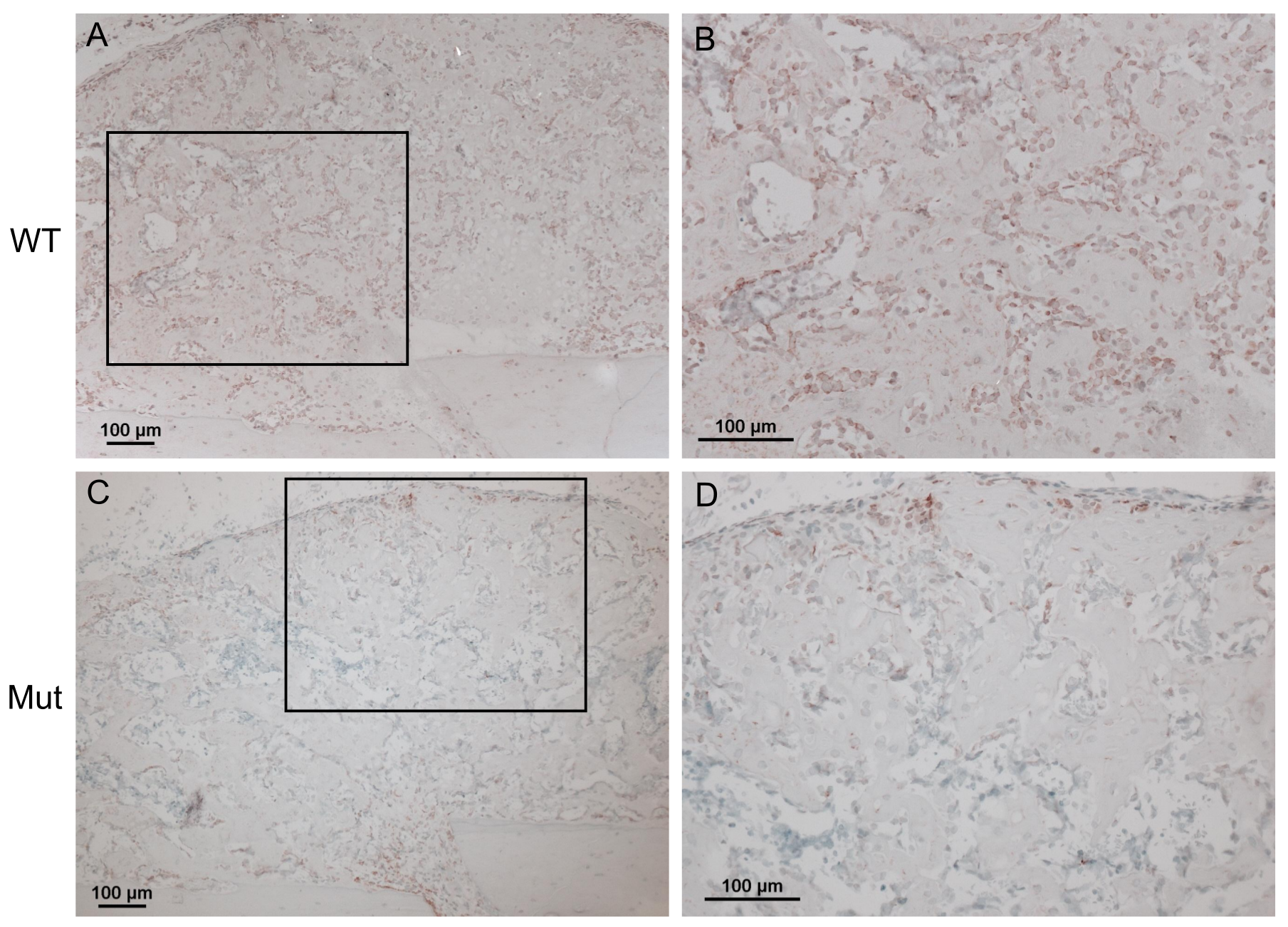

Supplement: Figure S12 — p-S6 IHC of fracture calluses at day 14 PF(A) 10× magnification of wild-type callus; (B) 20× magnification of box from (A); (C) 10× magnification of Pten mutant callus; (D) 20× magnification of box from (C). p-S6 was expressed at a similar level in each case. (TIF) [file pone.0063857.s012.tif]

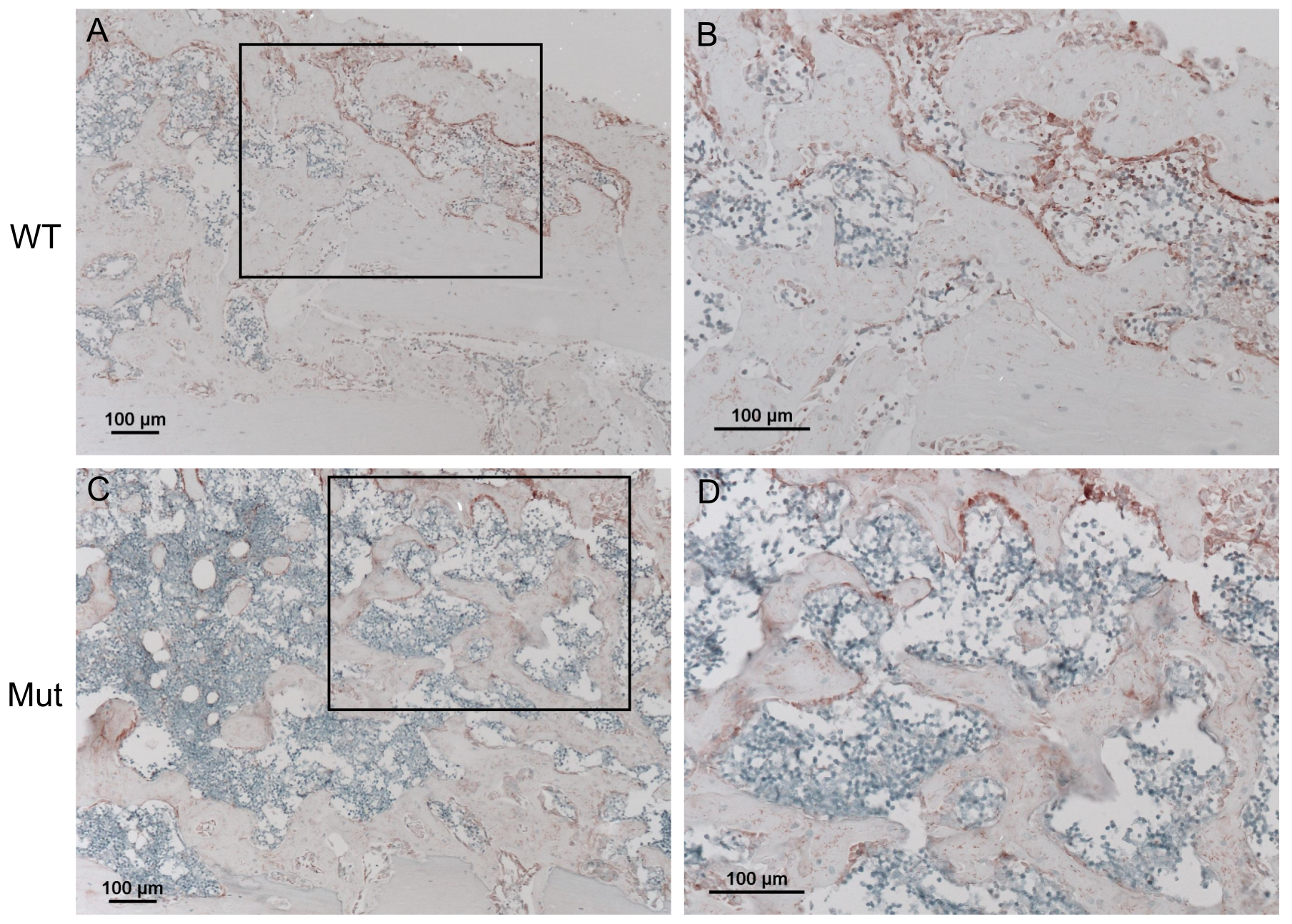

Supplement: Figure S13 — p-S6 IHC of fracture calluses at day 21 PF. (A) 10× magnification of wild-type callus; (B) 20× magnification of box from (A); (C) 10× magnification of Pten mutant callus; (D) 20× magnification of box from (C). p-S6 was expressed at a similar level in each case. (TIF) [file pone.0063857.s013.tif]

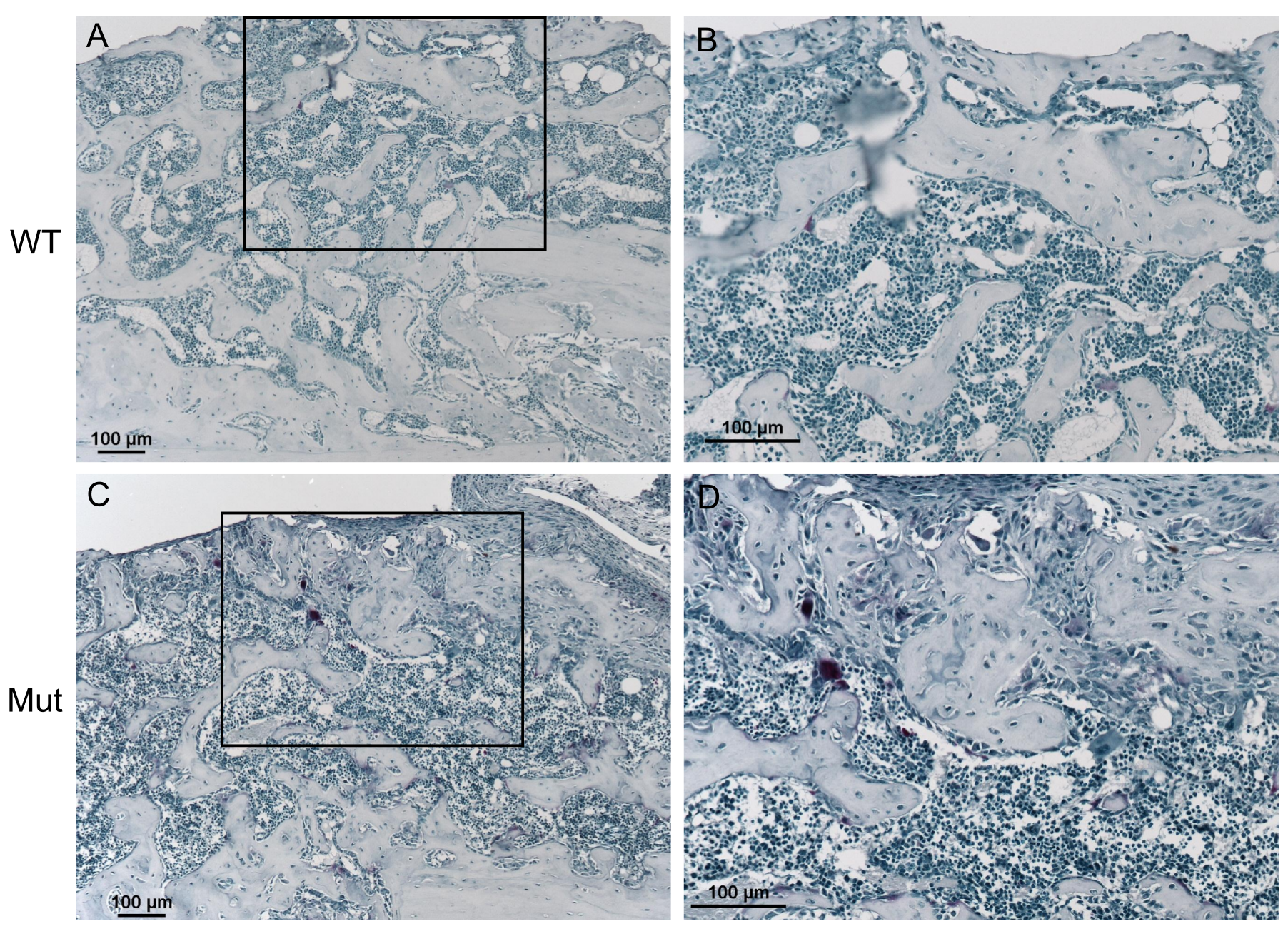

Supplement: Figure S14 — TRAP stain of fracture calluses at day 21 PF. (A) 10× magnification of wild-type callus; (B) 20× magnification of box from (A); (C) 10× magnification of Pten mutant callus; (D) 20× magnification of box from (C). TRAP staining was more intense in the mutant group. (TIF) [file pone.0063857.s014.tif]
