# Supplementary material for: Mice Lacking Pten in Osteoblasts Have Improved Intramembranous and Late Endochondral Fracture Healing
Source: PLoS One. 2013 May 13;8(5):e63857. doi: 10.1371/journal.pone.0063857 (PMC3652860; doi:10.1371/journal.pone.0063857)
Supplement: Document S1 — Supplemental methods. (DOCX) [file pone.0063857.s015.docx]

**Document S1: Supplemental Methods**

For immunohistochemistry, sections were deparaffinized and hydrated. Protease XXIV (P8038, Sigma-Aldrich, St. Louis, MO) was used for 30 minutes at 37 °C for antigen retrieval. Endogenous peroxidase was blocked with Immunopure (Fisher cat. no. 35000, Thermo Fisher Scientific Inc.,Waltham, MA) for 30 minutes in a humidified chamber at room temperature. Nonspecific binding was blocked by incubation in 10% normal goat serum in phosphate-buffered saline for 30 min in a humidified chamber at room temperature. Sections were incubated with primary antibodies (p-Akt: #3787, 1:50 dilution in 0.1% BSA, Cell Signaling Technology, Danvers, MA; p-S6, #4857, 1:75 dilution; Pten, #9559, 1:200 dilution) overnight in a humidified chamber at 4 °C. A biotinylated goat anti-rabbit secondary antibody (BA-1000, 1:200 dilution in 0.1% BSA, Vector Laboratories, Inc., Burlingame, CA) was used for 30 min in a humidified chamber at room temperature. Sections were stained using an avidin-biotin kit (Vectastain ABC kit PK-6100, Vector Laboratories, Inc.) followed by DAB-peroxidase (SK-4100, Vector Laboratories, Inc.). Sections were counterstained with hematoxylin (GHS3, Sigma-Aldrich) and mounted with Cytoseal 60 (8310-4, Thermo Fisher Scientific). For each primary antibody, a negative control was used by adding primary diluent without primary antibody to the section.
